# Lower Bounds for Nonrelativistic Atomic Energies

**DOI:** 10.1021/acsphyschemau.1c00018

**Published:** 2021-09-20

**Authors:** Robbie T. Ireland, Peter Jeszenszki, Edit Mátyus, Rocco Martinazzo, Miklos Ronto, Eli Pollak

**Affiliations:** † Institute of Chemistry, ELTE, Eötvös Loránd University, Pázmány Péter sétány 1/A, Budapest, H-1117, Hungary; # School of Chemistry, University of Glasgow, University Avenue, G12 8QQ, Glasgow, United Kingdom; ¶ Department of Chemistry, University of Milan, Milan, 20122, Italy; ⊥ Institute of Molecular Science and Technologies (ISTM), Consiglio Nazionale delle Ricerche (CNR), Milan, 20133, Italy; § Chemical and Biological Physics Department, Weizmann Institute of Science, 76100, Rehovot, Israel

**Keywords:** lower bounds, atomic energies, explicitly correlated Gaussians, two- and three-electron atoms, Cauchy−Schwartz inequality

## Abstract

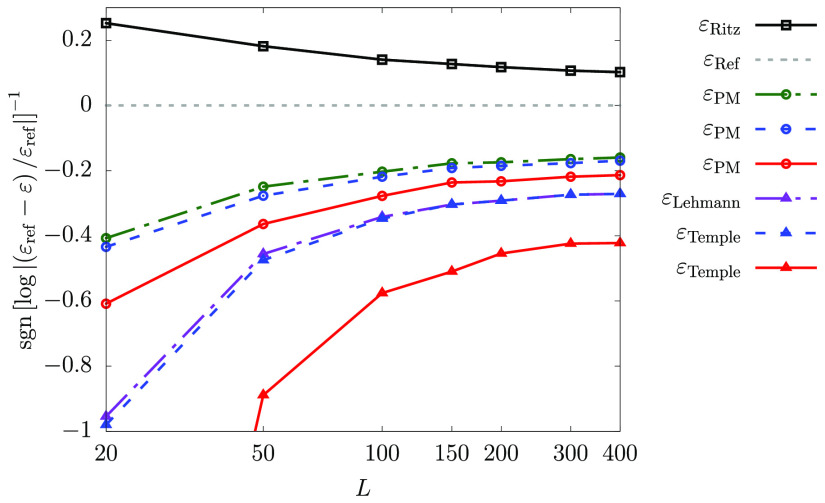

A recently developed lower bound theory for Coulombic problems (E. Pollak, R. Martinazzo, *J. Chem. Theory Comput.***2021**, *17*, 1535) is further developed and applied to the highly accurate calculation of the ground-state energy of two- (He, Li^+^, and H^–^) and three- (Li) electron atoms. The method has been implemented with explicitly correlated many-particle basis sets of Gaussian type, on the basis of the highly accurate (Ritz) upper bounds they can provide with relatively small numbers of functions. The use of explicitly correlated Gaussians is developed further for computing the variances, and the necessary modifications are here discussed. The computed lower bounds are of submilli-Hartree (parts per million relative) precision and for Li represent the best lower bounds ever obtained. Although not yet as accurate as the corresponding (Ritz) upper bounds, the computed bounds are orders of magnitude tighter than those obtained with other lower bound methods, thereby demonstrating that the proposed method is viable for lower bound calculations in quantum chemistry applications. Among several aspects, the optimization of the wave function is shown to play a key role for both the optimal solution of the lower bound problem and the internal check of the theory.

## Introduction

The challenge of obtaining lower bounds for atomic and molecular energies has a long history. In 1928, Temple^1^ derived the first and relatively general expression for obtaining lower bounds to ground state energy eigenvalues. In contrast to the Ritz method,^2^ which gives upper bounds, Temple’s lower bound expression is mathematically more complex and computationally more demanding. It requires the evaluation of not only the Ritz eigenvalue, (λ_*j*_), but also the variance (σ_λ_*j*__^2^) associated with the trial wave function, as well as some information on the energy of an excited state. Hence, for the evaluation of Temple’s lower bound, the Hamiltonian squared matrix is necessary. Intuitively, one may expect that upper and lower bounds for the *j*th exact eigenvalue, ε_*j*_, are provided by λ_*j*_ ± σ_λ_*j*__. Indeed, Weinstein^3^ showed in 1934 that the ε_*j*_ ≥ λ_*j*_ – σ_λ_*j*__ lower-bound condition holds for the *j*th exact eigenvalue under certain conditions. This result was refined by Stevenson^4^ four years later. These important results underlie the formal mathematical proof for the stability of an atom when described in quantum mechanics,^5,6^ but are less useful in numerical computations. The Temple and Weinstein–Stevenson expressions result in “poor” numerical lower bounds in the sense that the gap ratio, the ratio of the deviation of the upper and the lower bound from the exact value, (ε_*j*_ – ε_*j*,–_)/(ε_*j*,+_ – ε_*j*_), is all too often orders of magnitude larger than unity. (Lower and upper bounds are denoted with – and + subscripts respectively throughout.)

Why should one care? Is it not enough to have accurate upper bounds generated by the Ritz procedure? In this context, we note for example, that atomic and molecular physics are going through a renaissance period thanks to the rapid development of precision spectroscopy of light atomic and molecular systems. The experimental uncertainty of several measured transition (and dissociation) energies of small systems have been reduced by orders of magnitude over the past decade.^7−11^ When a good agreement is found between experiment and theory, it can be used for refinement of fundamental constants and quantities (e.g., the Rydberg constant, the electron-to-proton or electron-to-nucleus mass ratio), if the experimental and theoretical uncertainties are comparable. It is therefore important to have rigorous theoretical error bars that can be computed for the (transition) energies. Such error bars do not exist when considering energy differences without having both upper and lower bounds to the energy eigenvalues of the states involved in the transition. The same is true when considering tunneling splittings between adjacent levels.^12^ Rigorous energy upper bounds can be computed for increasingly comprehensive levels of the atomic and molecular theory, that is, for the (a) nonrelativistic electronic energy corresponding to an infinite nuclear mass, for the (b) “pre-Born–Oppenheimer” energy with all electrons and atomic nuclei included in the quantum system,^13,14^ and even for (c) the no-pair, many-particle Dirac energy with (retarded) electromagnetic interaction.^15,16^

Theoretical error bars to the computed upper bound energies are typically estimated from inferring the convergence (rate) of the upper bound with respect to the length of some basis set expansion. These “empirically” inferred error bars sometimes turn out to be overly optimistic. Apart from the inherently theoretical questions, these considerations underline the practical need for an accurate and readily implementable lower bound theory.

This situation presents an intriguing challenge. Both the Temple and the Weinstein lower bounds were typically implemented using the following strategy. First, one sets up the Hamiltonian matrix, say with a basis set of dimensionality *L*. Finding the “best” estimate is then obtained by diagonalizing the matrix and, owing to the variational theorem,^17^ one is guaranteed that λ_*j*_ ≥ ε_*j*_, *j* = 1, ..., *L*. The diagonalization of the matrix, which is at the heart of the Ritz method, finds the linear combination of basis functions which gives the least upper bound. One might then pose the question: what is the linear combination of basis functions which gives the largest lower bound? The answer was given by Lehmann in 1949.^18,19^ He showed that minimization of the resolvent of the Hamiltonian using the Ritz method gives a maximal Temple-class lower bound. The practical application of Lehmann’s method does not require the computation of the resolvent, but Lehmann’s equation contains the matrices of the Hamiltonian and the Hamiltonian squared.^20^

Lehmann’s method was a significant improvement over Temple’s result, yet, as far as atomic energies were concerned, the actual lower bounds obtained even when using Lehmann’s method, were not very good. In 1994, Lüchow and Kleindienst,^21^ using 920 Hylleraas-type basis functions^22^ and Lehmann’s method, were able to converge from below the ground-state energy of the lithium atom with an accuracy of ∼10^–4^ E_h_ (15 ppm (parts per million) relative precision).

This challenge has also led to other, innovative methods of obtaining lower bounds. For atoms, a central source of difficulty is the electronic Coulomb repulsion term (1/*r*_12_). Fortunately, this term is positive so that subtracting it from the Hamiltonian results in a separable sum of hydrogen-like Hamiltonians, the solution of which is known analytically and which are themselves (poor) lower bounds to the exact energies. Bazley^23,24^ and then Bazley and Fox,^25^ improving upon the suggestion of Aronszajn,^26^ noted that the Cauchy–Schwartz inequality implies that the inverse of the mean of *r*_12_ is always less than the mean of 1/*r*_12_ so that one may construct a series of approximate Hamiltonians (bracketing functions), all of which are less than the original Hamiltonian. Their energies bound the exact eigenvalues from below. Further analysis and improvements upon Bazley’s method as well as comparisons with Temple-based lower bounds have been presented by Marmorino.^27−29^ However, here too the bottom line is not very encouraging. The complete basis set of the “base” separable Hamiltonian is not complete for a multielectron atom so that the method will not necessarily converge to the exact answer. Although Bazley’s method and its variants have their uses, it too is not of sufficient generality and accuracy.

Löwdin^30^ improved upon the Aronszajn–Bazley–Fox approach by considering a different bracketing function based on the resolvent, that is the matrix inverse of *E* – *H* (*H* denoting the Hamiltonian operator). However, as noted by Szabados and Toth,^31,32^ it is not widely used due to the need to actually compute the inverse. Miller^33^ further improved the methodology by bounding the inverse from below, in a way similar to the Bazley–Fox use of the Cauchy–Schwartz inequality. However, the approach suffers from the fact that, as noted by Marmorino in his Ph.D. thesis,^34^ it becomes very costly as the number of electrons increases since the computation cannot be expressed in terms of only two electron integrals, but will involve full *N*-electron integrals.

Perhaps the most impressive application of Temple lower bounds was reported by Naka-shima and Nakatsuji^35^ who computed the Ritz upper bound to a 40-digit accuracy. The accuracy of their lower bound computations was worse by more than 10 orders of magnitude.

There are a few difficulties associated with the Lehmann class of lower bound methods. First, the poor convergence has to do with the fact that variances converge much slower with increasing dimensionality than means. This has been discussed at some length by Caldow and Coulson.^36^ As noted in ref (35), when computing the variance, the integrand of the *H*^2^ matrix element is positive so that all errors in the wave function contribute to the error in the computation of the matrix element. In contrast, when computing the matrix element of *H*, positive and negative errors tend to cancel each other out, leading to improved convergence. Second, input is needed in the form of known excited state energies or lower bounds to them. These are also needed in the Lehmann form, where the computation of the “Lehmann pole”^20^ is not trivial. Third, the actual computation of the matrix of *H*^2^ is computationally costly.

These difficulties have been addressed in a recent series of papers.^12,37−42^ A central aspect which has led to significant improvement is combining lower bound theory with basis sets created by the Lanczos method.^43^ Due to the resulting tridiagonal representation of the Hamiltonian the computation of the variance needs as input only matrix elements of the Hamiltonian itself. This is perhaps not surprising, as the Lanczos basis set depends on the Krylov basis,^44^ which contains the series of powers of the Hamiltonian. Thus, the normalization of each added Lanczos function implicitly has in it the variance associated in the previous one. A more profound simplification is that when using the Lanczos basis, there is no need to diagonalize the *H*^2^ matrix separately. It is sufficient to know all the Ritz eigenvalues and their associated variances, and these are readily obtained through the Lanczos construct. The diagonalization of the Lehmann equation becomes equivalent to the solution for the zeros of a sum of rational terms. Third, our recent self-consistent lower bound theory^40,42^ leads to gap ratios which are of the order of unity, and sometimes even less.

The trouble is that the Lanczos basis set is irrelevant when it comes to Coulombic systems, as the Coulomb term leads to divergences of matrix elements of the third and higher powers of the Hamiltonian. To overcome this difficulty, we have recently shown^41^ that the formal eigenvalue equation derived from the Lehmann equation can be used to derive lower bounds, even when the Lanczos method is invalid. The accuracy of this new method can compete with that of the Ritz upper bounds. This “Pollak–Martinazzo” (PM) theory was applied to the hydrogen and helium atoms to both ground and a few excited states. Yet, especially for He, we used a limited seven-dimensional basis set, so that the absolute accuracy of the upper and lower bounds to the ground state was in the milli-Hartree range only and not enough for what is considered to be chemical accuracy, that is in the sub-milli-Hartree range.

This paper is devoted to further development and implementation of the PM method to helium and helium-like two electron ions, as well as to the lithium atom, using explicitly correlated Gaussian (ECG) basis sets.^13,45^ We use up to 400 functions for two-electron atoms and 900 functions for Li, for which the resulting Ritz ground state eigenvalue precision is on the parts-per-billion (ppb) level. The employment of ECG basis sets compared to, for example, Hylleraas basis sets, is motivated by the highly accurate upper bounds, of the order of ppb’s, which is obtained with a relatively small number—of the order of 10^3^ basis functions—and its applicability to molecular systems. This in return is very useful for answering the challenges presented by high precision atomic and molecular spectroscopy.

The numerical implementation of ECG basis sets and the associated optimization of the parameters of the Gaussians is described in some detail in refs (13, 14, 45, and 46). A central obstacle in the application of PM theory is the need to compute variances. This becomes especially difficult when using correlated Gaussian basis sets due to the Coulomb singularity. The necessary integrals have been reported in ref (47) (see also the Supporting Information to this work). Using this newly developed methodology, the PM theory gives lower bounds with subparts-per-million (ppm) precision. As far as we could ascertain from the literature on the ground state of the Li atom, the lower bound reported in this paper is the best lower bound ever obtained. Not yet as tight as the upper bounds, yet an important step forward in providing numerical algorithms for lower bounds for atomic energies, which are of chemical accuracy.

In the second section of this paper we briefly review the Temple, Lehmann, and PM lower bound theories and extend the latter so that it may be used in conjunction with eigenfunctions of Lehmann’s equation and the associated diagonal matrix elements and variances. The correlated Gaussian basis set and the computation of variances using it is described in the third section. Results for the He, Li^+^, H^–^, and Li atomic systems are given in the fourth section. We end this paper with a discussion, considering future prospects and improvements to PM theory, paying special attention to applications with explicitly correlated Gaussian basis sets.

## Lower Bound Theory Based on Temple’s Work

### Preliminaries

The notation we use is that of a Hamiltonian operator with eigenstates and eigenvalues in ascending order:
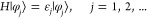
2.1In a typical computation one starts with some known orthonormal basis set |Ψ_*j*_⟩, *j* = 1, 2, ... which is assumed to span the full Hilbert space of the Hamiltonian so that the identity operator is
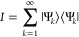
2.2The Hamiltonian operator may be therefore represented as
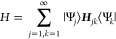
2.3with the notation
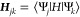
2.4for the matrix elements of the operator in the chosen basis set.

In practice, one is limited to a finite basis set, with say *L* states spanning a subspace . The projector onto this finite space is by definition
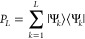
2.5and its orthogonal complement is denoted as *Q*_*L*_ such that *P*_*L*_ + *Q*_*L*_ = *I*. The projected Hamiltonian is
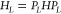
2.6and since it is finite, it may be diagonalized to give the Ritz eigenvalues λ_*j*_^(*L*)^ with associated normalized eigenfunctions |Φ_*j*_^(*L*)^⟩
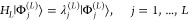
2.7We denote the overlap squared (for the sake of brevity we assume real functions everywhere) of the *j*th eigenfunction in the projected space with the exact *k*th eigenfunction as
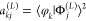
2.8The variance (σ_*j*_^(*L*)^)^2^ associated with the *j*th eigenfunction of the projected Hamiltonian is
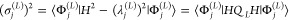
2.9The second equality may pave the way for computing variances without necessitating the computation of matrix elements of *H*^2^.

### The Weinstein Lower Bound

A central element of lower bound theory is a Cauchy–Schwartz inequality, valid for any projection operator *Q* and state |Ψ⟩
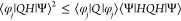
2.10We first choose the projection operator to be
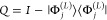
2.11Then, by replacing |Ψ⟩ in eq 2.10 with the Ritz eigenfunction |Φ_*j*_^(*L*)^⟩ and using the second equality in eq 2.9 for the variance, which remains valid if we replace *Q*_*L*_ with the projection operator of eq 2.11, we can manipulate the Cauchy–Schwartz inequality so that it gives a lower bound expression to the *j*th eigenvalue as
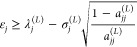
2.12which at this point is not very useful since we do not know the overlap *a*_*jj*_^(*L*)^.

If we know that our choice of basis set is sufficiently “good” such that we may assume that *a*_*jj*_^(*L*)^ ≥ ^1^/_2_, then eq 2.12 immediately gives the Weinstein lower bound expression
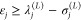
2.13It turns out that the assumption that *a*_*jj*_^(*L*)^ ≥ 1/2 is less restrictive^40^ than Stevenson’s^4^ condition of validity that the Ritz eigenvalue λ_*j*_^(*L*)^ is the closest one to the true eigenvalue ε_*j*_:
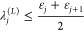
2.14

### Temple’s Lower Bound

The derivation of Temple’s lower bound is slightly more involved. With each Ritz eigenvalue one introduces a “residual energy” λ̅_*j*_^(*L*)^ defined as
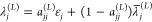
2.15which implies that
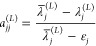
2.16Inserting this result into the inequality of eq 2.12 and rearranging gives the Temple lower bound expression
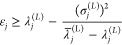
2.17where the previous unknown overlap *a*_*jj*_^(*L*)^ has been replaced by the residual energy. The advantage of this manipulation is that from its definition (eq 2.15) one may rewrite the residual energy in terms of the overlaps and the exact eigenenergies as
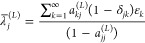
2.18where δ_*jk*_ is the Kronecker delta. Equation 2.18 may be used to obtain lower bounds to the residual energy.^40,42^ For example, for the ground state we can write
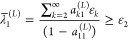
2.19so that the Temple lower bound for the ground state energy may be expressed as
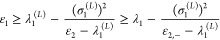
2.20where ε_2,–_ denotes a lower bound to the first excited state energy which can be obtained by other means, such as by using the Weinstein- or the Bazley-type lower bounds. Indeed, for the He atom, Bazley already found a lower bound energy for the first excited state of −2.165 E_h_^23^ to be compared with the numerically exact energy of −2.146 E_h_. Although the lower bound is not of chemical accuracy, it is sufficiently tight in the sense that (ε_2,–_ – ε_1_)/(ε_2_ – ε_1_) ≃ 0.97, so it would barely change the quality of the resulting Temple lower bound, especially if λ_1_^(*L*)^ is a “good” upper bound. Obtaining lower bounds to the residual energy of excited states is more involved, though straightforward when using a Lanczos basis set. A detailed derivation and discussion may be found in refs (40−42).

### Lehmann’s Lower Bound Theory

As discussed in the Introduction, Temple’s lower bound expression as derived above uses the Ritz eigenvalues and eigenfunctions. Lehmann’s theory parallels the Ritz theory in the sense that within the subspace  it leads to the linear combination which maximizes Temple’s lower bound. First one introduces the “Lehmann pole” ρ which may be any real number, excluding the Ritz eigenvalues. Within the projected space, there will be some Ritz eigenvalue, say with index L* < *L* such that one knows that λ_L_^*(*L*)^ ≤ ε_L*+1_. The interleaving theorem then assures us that for all *j* ≤ L*, λ_*j*_^(*L*)^ ≤ ε_*j*+1_. The Lehmann pole ρ is chosen such that it obeys the inequality λ_L_^*^ < ρ ≤ ε_L*+1_. Lehmann’s optimizing equation is
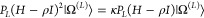
2.21The Lehmann eigenvalues κ and eigenfunctions  are readily found by diagonalizing the equation. The condition on the Lehmann pole then assures us that one will have L* negative eigenvalues and the associated values τ = κ + ρ will be the optimal Temple lower bounds for the first L* ≤ *L* eigenvalues. The numerical implementation is then to choose the Lehmann pole as a lower bound to the energy ε_L*+1_ which is higher than λ_L*_^(*L*)^. This is not a trivial demand, but lies at the heart of the Lehmann algorithm. In practice, such a lower bound may be obtained via the Weinstein- or Bazley-type lower bounds, but even this procedure is not trivial.

It is straightforward to understand why Lehmann’s equation leads to optimal lower bounds. Consider the (unnormalized) vector
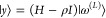
2.22defined for any vector . Lehmann’s equation is then equivalent to the stationary condition of the Rayleigh–Ritz quotient involving the resolvent *G*(ρ) = (*H* – *ρI*)^−1^:
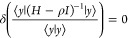
2.23The solution of this variational problem is the vector |*Y*^(*L*)^⟩ = (*H* – *ρI*)|Ω^(*L*)^⟩ resulting from the Lehmann eigenfunction |Ω^(*L*)^⟩. The eigenvalue κ is then
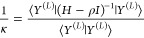
2.24demonstrating that κ^–1^ is a Ritz eigenvalue for the resolvent *G*(ρ) = (*H* – *ρI*)^−1^. The interleaving theorem (Courant–Fischer theorem) assures us that the negative values κ^–1^ bound the exact eigenvalues (ε_*k*_–ρ)^−1^ of *G*(ρ) from above, provided that ε_*k*_ is lower than ρ. Sorting the negative κ values in order of decreasing magnitude, |κ_L*_| ≤ |κ_L*–1_| ≤ ...|κ_1_|, implies that τ_*n*_ = ρ + κ_*n*_ is a lower bound to the (L* – *n* + 1)th eigenvalue left of ρ. The lower bounds are therefore ordered as τ_1_ ≤ ε_1_; τ_2_ ≤ ε_2_; ...; τ_L*_ ≤ ρ. Finally, multiplying eq 2.21 with the bra ⟨Ω^(*L*)^| and rearranging, one finds that
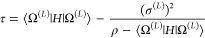
2.25where (σ^(*L*)^)^2^ = ⟨Ω^(*L*)^|*H*^2^|Ω^(*L*)^⟩ – ⟨Ω^(*L*)^|*H*|Ω^(*L*)^⟩^2^ is the energy variance associated with the Lehmann eigenfunction |Ω^(*L*)^⟩, demonstrating that Lehmann’s lower bound is indeed of Temple form.

Lehmann’s eigenvalue equation takes an especially simple form when the basis set is of Lanczos type. In this case, the variance as obtained from eq 2.9 takes the form
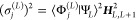
2.26At the same time, multiplying Lehmann’s equation, eq 2.21, from the left by the bra ⟨Φ_*k*_^(*L*)^| and rearranging, using the Lanczos tridiagonal property one finds
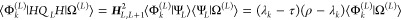
2.27Squaring the equality on the right-hand side and summing over all *k* from 1 to *L* gives an eigenvalue equation, which is explicitly based on the Lanczos construct
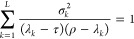
2.28This implies that, for a Lanczos basis set, one does not need to explicitly diagonalize the Lehmann equation as it suffices to find the lowest L* solutions of this equation to obtain the lower bounds. The input consists of the Ritz eigenvalues and eigenvectors and the Hamiltonian matrix element ***H***_*L*,*L*+1_, considerably simplifying the theory. However, one does remain with the need to estimate the Lehmann pole.

### PM Lower Bound Theory

The challenge is to generalize the Lehmann equation valid for the Lanczos basis set, even when one cannot set up such a basis set, such as in Coulombic systems due to the divergence of cubic and higher moments of the Hamiltonian. For this purpose we construct a diagonal matrix “Hamiltonian” of dimensionality (*L* + 1) × (*L* + 1) which has the form
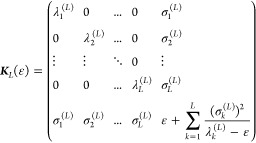
2.29where ε is an energy value to be chosen according to the eigenvalue one wants to bound from below. One then readily finds that ε is an eigenvalue of this matrix Hamiltonian and the rest of the eigenvalues, denoted by *x*, are the *L* solutions of the polynomial equation
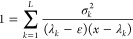
2.30which is identical in form to eq 2.28 with the identification τ → ε and ρ → *x*. A straightforward analysis of this equation shows that , which implies that any solution *x* is a monotonically increasing function of the energy parameter ε. For the interested reader, a detailed analysis of this and additional properties is given in ref (41). Suffice it here to say that the *k*th root of the equation, *x*_*k*_(ε), is continuous on the real axis except for a single pole singularity at a Ritz eigenvalue λ. It is monotonically increasing as a function of ε in the two subdomains (−∞, λ), (λ, +∞).

Consider then the ground state, where we choose the energy parameter to be equal to the (unknown) true ground-state energy (ε = ε_1_). We may order the *L* remaining poles of eq 2.30 in ascending order. Suppose we have a lower bound to the lowest pole *x*_1_^(*L*)^(ε_1_), call it *x*_1,–_. Inserting this lower bound into eq 2.30 we find the lowest root ε_1,–_^(*L*)^ which, due to the monotonicity property, must be lower than ε_1_ and therefore a lower bound to it. The key to obtaining a lower bound to the ground state energy is to find a lower bound to *x*_1_^(*L*)^(ε_1_). In general terms, due to the interleaving theorem and the way we constructed the matrix Hamiltonian, we know that λ_1_^(*L*)^ ≤ *x*_1_^(*L*)^(ε_1_) ≤ λ_2_^(*L*)^. Necessarily then, when the basis set is sufficiently “good”, *x*_1_^(*L*)^(ε_1_) will be close to, but below, λ_2_^(*L*)^. Moreover, one may follow, at least in principle, how *x*_1_^(*L*)^(ε) changes with increasing *L* for any value of ε close to the exact ground state energy value. If it is a decreasing function, then necessarily it will be larger than the first excited state energy ε_2_, since both the Ritz eigenvalue λ_2_^(*L*)^ → ε_2_ and *x*_1_^(*L*)^(ε_1_) → ε_2_ when *L* becomes large enough.

This strategy may then be used also for excited states. For example, if the basis set is accurate enough so that *x*_2_^(*L*)^ ≥ ε_3_, then inserting ε_3_ into eq 2.30 gives lower bounds to the ground and first excited state energies, and this process may be continued to higher lying states. One notices that the challenge of finding a lower bound to *x*_*j*_^(*L*)^(ε_*k*_) is very similar to the challenge of finding the appropriate Lehmann pole.

One may further generalize the PM method. Given a basis set of finite dimension *L*, in anticipation of the practical implementation in the context of the Lehmann eigenvectors denoted as |Ω_*j*_^(*L*)^⟩, *j* = 1, ···, *L* one may construct diagonal elements and associated variances as
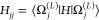
2.31
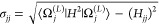
2.32The *H*_*jj*_ matrix elements are then arranged in ascending order, and one may construct with them a matrix Hamiltonian just as in eq 2.29, by replacing the Ritz eigenvalues and associated variances with the *H*_*jj*_ and associated σ_*jj*_ values. The PM equation, eq 2.30, remains the same, the main difference is that, apart from the ground-state matrix element *H*_11_ (and all other diagonal matrix elements) which is always greater than or equal to the true ground-state energy, it is no longer necessarily the case that all other diagonal elements bound the excited state eigenvalues from above. This makes it more difficult to ascertain that the condition *x*_*k*_(ε_1_) ≥ ε_*k*+1_ holds. However, the fact that *H*_11_ ≥ ε_1_ assures that if one can show that *x*_1_(ε_1_) ≥ ε_2_, then using ε_2_ in eq 2.30 guarantees that *x*_1_(ε_2_) is a lower bound to the true ground-state energy. In practice, if one knows the approximate vicinity of the ground-state energy, one may study the dependence of *x*_1_(ε) in this vicinity and if one finds that it is greater or equal to the excited-state energy ε_2_, then by inserting ε_2_ into eq 2.30 one is assured that the resulting ground-state pole *x*_1_(ε_2_) is a lower bound to the ground-state energy. This process may then be continued also for excited states.

To summarize, the PM method has a few advantages as compared to previous theory. In principle, when using the Ritz eigenvalue basis set, there is no need to compute the full Hamiltonian squared matrix, as it suffices to obtain the Ritz eigenvalues and variances. In all applications thus far, the resulting lower bounds were found to be superior to any other method. However, there does remain the need to have information on excited-state energies to obtain lower bounds for energies lying below them.

## Explicitly Correlated Gaussian Basis Sets

Explicitly correlated Gaussian (ECG) basis sets^13,45,48−51^ are useful for highly accurate energy computations of atoms and molecules. They explicitly account for pair correlation and allow for a rapid convergence of the energy with the basis set size, while the integrals needed to compute the Hamiltonian and overlap matrices can be evaluated in a closed, analytic form. It is necessary to mention that ECGs fail to describe the exact behavior of the nonrelativistic wave function at the particle–particle coalescence points^52,53^ and for large particle separations. For almost all lower bound implementations, matrix elements are required not only for the nonrelativistic Hamiltonian, *H*, but also for the Hamiltonian squared, *H*^2^. The latter contains additional terms, sometimes referred to as “singular terms,” which must be calculated. For atomic and some small molecular computations, the Hylleraas-type basis^22,54^ is a good choice as these functions naturally simplify the computation by removing the Coulomb poles. Nevertheless, having molecular computations in mind (for the longer term), we present the first “real-life,” (sub)chemical accuracy applications (and numerical observations) for the PM theory, for the example of two- and three-electron atoms and ions as obtained with an ECG basis. The ECG integrals necessary for the evaluation of the *H*^2^ expectation values are considered in detail and some integrals that were not yet available in the “integral library” of the in-house developed QUANTEN computer program^55^ are reported in ref (47).

The Schrödinger equation, eq 2.1, is solved using a finite basis expansion in terms of ECG basis functions ψ_*i*_, defined as
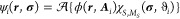
3.1where  is the antisymmetrization operator, **σ** denotes the spin variables, and ϑ_*i*_ is a parameter defined below. In this work, which focuses on the computation of the ground electronic state of atoms and atomic ions, the ϕ spatial ECG is centered at the origin (where the nucleus is fixed),
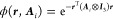
3.2where  collects the electron coordinates, ***I***_3_ is the three-dimensional unit matrix, and ϕ(***r***, ***A***_*i*_) is parametrized through the  positive-definite, symmetric matrix of spatial width coefficients. Since the ECGs are not orthogonal, the Hamiltonian matrix equation, to be diagonalized for *L* functions, takes the form
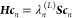
3.3where the matrices are defined as
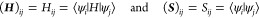
3.4The λ_*n*_^(*L*)^ eigenvalue, also called the Ritz energy, is an upper bound to the *n*th exact eigenvalue of *H*.

In the present work we consider the nonrelativistic Hamiltonian, which is spin-independent and thus commutes with the total spin operator, *S*^2^, and its projection on an axis, *S*_*z*_. Thereby, the fundamental structure of the χ_*S*,*MS*_ spin function is obtained as the eigenfunction of the spin operators, characterized by the spin quantum numbers.^56^ The singlet (*S* = 0, *M*_*S*_ = 0) spin function for two electrons (*n*_p_ = 2), relevant for the helium atom and helium-like ions, is
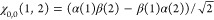
3.5The doublet spin-state (*S* = 1/2 with the +1/2 spin projection, for example) for the three-electron problem (*n*_p_ = 3) of lithium forms a doubly degenerate subspace. Hence, an additional free parameter, ϑ_*i*_, has to be introduced to ensure the complete description of the spin part,^45,46^
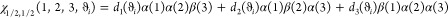
3.6

The ***c***_*n*_, ***A***_*i*_, and ϑ_*i*_ parameters are selected by minimization of the Ritz energy. At first glance, the optimization of the ϑ_*i*_ parameter may appear unnecessary, since the nonrelativistic Hamiltonian is spin-independent. We note that the spin and spatial parts of the wave function in eq 3.1 are entangled through antisymmetrization. In practical terms, this means that simultaneous optimization of *c*_*i*_ and ***A***_*i*_, as well as ϑ_*i*_ is necessary to arrive at an (near) optimal overall parametrization.

For spin-independent operators, the matrix representation of the Hamiltonian in eq 3.4 can be constructed if the matrix elements are known for the ϕ spatial basis functions. For the implementation of the considered lower bound theories, it is necessary to calculate the matrix elements of the *H*^2^ Hamiltonian squared operator,
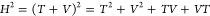
3.7where *T* and *V* are the kinetic and potential energy operators, respectively. The ECG integrals needed for the *T*^2^, *TV*, and *VT* operators are closely related to those appearing in the expectation value of the Breit–Pauli Hamiltonian (perturbative relativistic corrections)^57,58^ and have already been used in the literature^59,60^ and in the QUANTEN computer program.^16,55,61−63^ The calculation of the *V*^2^ matrix elements is newly implemented in this work (the integral expressions are summarized in the Supporting Information, see also ref (47)).

The basis function parameters were optimized using a stochastic (energy) minimization approach^45^ that was followed by repeated refinement cycles using the Powell method.^64^ For an accurate evaluation of the *H*^2^ matrix elements, we used an increased precision arithmetic (16-byte reals, quadruple precision in Fortran).

## Computations for two- And Three-Electron Atomic Systems Using Explicitly Correlated Gaussian Basis Sets

### Lower Bounds Using Energy-Optimized Basis Sets

**Figure 1:**
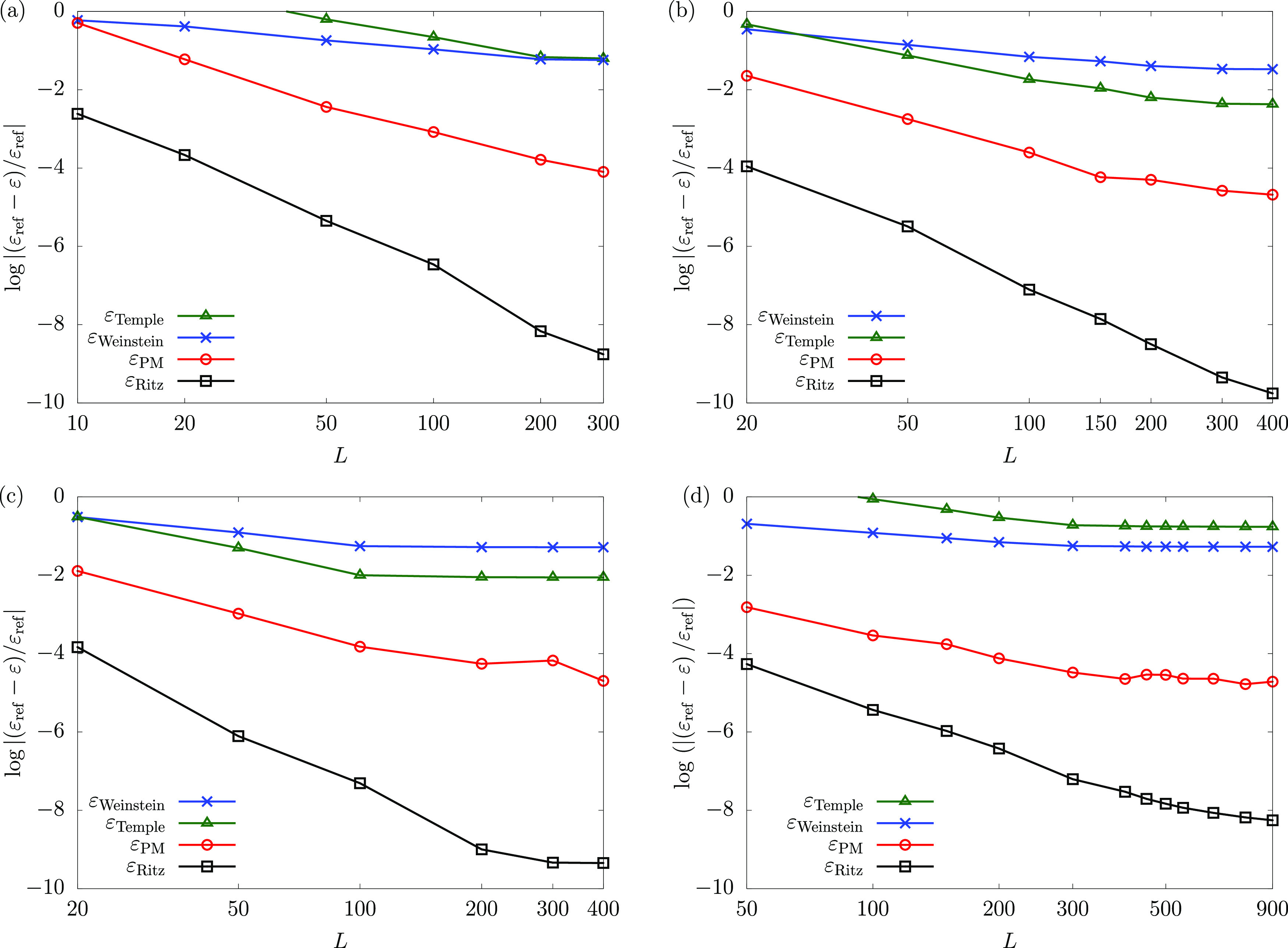
Convergence of the Ritz upper bound and the Weinstein, Temple, and PM lower bound energies for the ground states of (a) H^–^, (b) He, (c) Li^+^, and (d) Li with respect to the basis set size. The basis functions were generated and refined based on the energy minimization criterion of the Ritz upper bound to the ground state energy. The ε_ref_ reference ground-state energies (converged to 32 digits for the helium atom) are taken from refs (35, 65, and 66).

The lower bounds of Temple, Weinstein, and Pollak–Martinazzo (PM) were first applied to multiple systems using an ECG basis set optimized to minimize the ground state Ritz energy of the system. Figure 1 shows the upper and lower bound energies calculated for the two-electron H^–^, He, and Li^+^ atomic systems, as well as the three-electron Li atom. The numerical values of the best lower bounds are given in Table 1. In this work, we have assumed an infinite mass for the nucleus. We only note here that this assumption can be lifted and the current work can be generalized to include all particles in the quantum system.^14,46^

**Table 1:** Energy Bounds, in E_h_, to the Ground-State Energy of H^–^, He, Li^+^, and Li Shown in Figure1 Panels a, b, c, and d, respectively, Obtained Using Energy-Optimized ECG Basis Sets^a^

energy bound	H^–^	He	Li^+^	Li
reference (Ritz)	–0.527 751 016 5^65^	–2.903 724 377 0^65^	–7.279 913 413^65^	–7.478 060 324^67^
Ritz	–0.527 751 015 6	–2.903 724 376 5	–7.279 913 409	–7.478 060 309
Temple	–0.560 886 142 4	–2.916 136 676	–7.344 289 285	–8.766 772 761
PM	–0.527 793 235 5	–2.903 784 830	–7.280 059 525	–7.478 184 715

a The λ_*j*_ and σ_*j*_ values for the lower-bound expressions were computed with the Ritz eigenvectors. We used ε_2_^–^ = −0.5 E_h_ and −5.040 876 8 E_h_ for H^–^ and Li^+^, respectively. For the ε_2_^–^ values of He and Li, see Table 2. Tables 3 and 4 report more elaborate results for He and Li, respectively.

Inspection of Figure 1 shows that among all energy estimates, the Ritz upper bound energy is by far the best-converged energy for all four systems. Of the studied lower bound estimates the PM lower bound energy is the tightest.

It is interesting that for the hydride ion and the lithium atom, the Weinstein lower bound energy is more accurate than Temple’s lower bound. This can be understood by considering the denominator of eqs 2.20 and 2.30, both of which contain the difference (λ–ε). If ε is taken to be the energy of the first-excited state (hydride has only one bound state, and so the hydrogen ground state energy may be used as ε_2_), then for the hydride ion and the lithium atom this difference is notably smaller than for the other systems. For hydride and the lithium atom, it is less than 0.3 E_h_, whereas for the helium atom the difference is approximately 0.8 E_h_, and for the lithium cation it is more than 2.0 E_h_. Thereby, the magnitude of the fraction itself is greater for the hydride and the lithium atom, and this results in worse Temple bounds. For Weinstein’s lower bound, eq 2.13, this difference is not present, and so it is the magnitude of the standard deviation that is of importance.

One will also notice the anomalous decrease in the PM lower bound with the increasing dimensionality of the subspace for the lithium ground state around 400–550 basis functions, and again around 900 basis functions. The reason for this behavior may stem from the fact that when using ECG basis functions, which are optimized to minimize the ground state Ritz energy (e.g., ref (45)), the PM lower bound is not necessarily maximized simultaneously, and so, it does not need to be a monotonically increasing function. This observation is addressed later in this section (Figure 8).

After analyzing these data sets, efforts were focused on methods which would improve the accuracy of the lower bounds. In the following subsections, we discuss the implementation of various options that improved upon the initial results (Figure 1).

**Figure 2:**
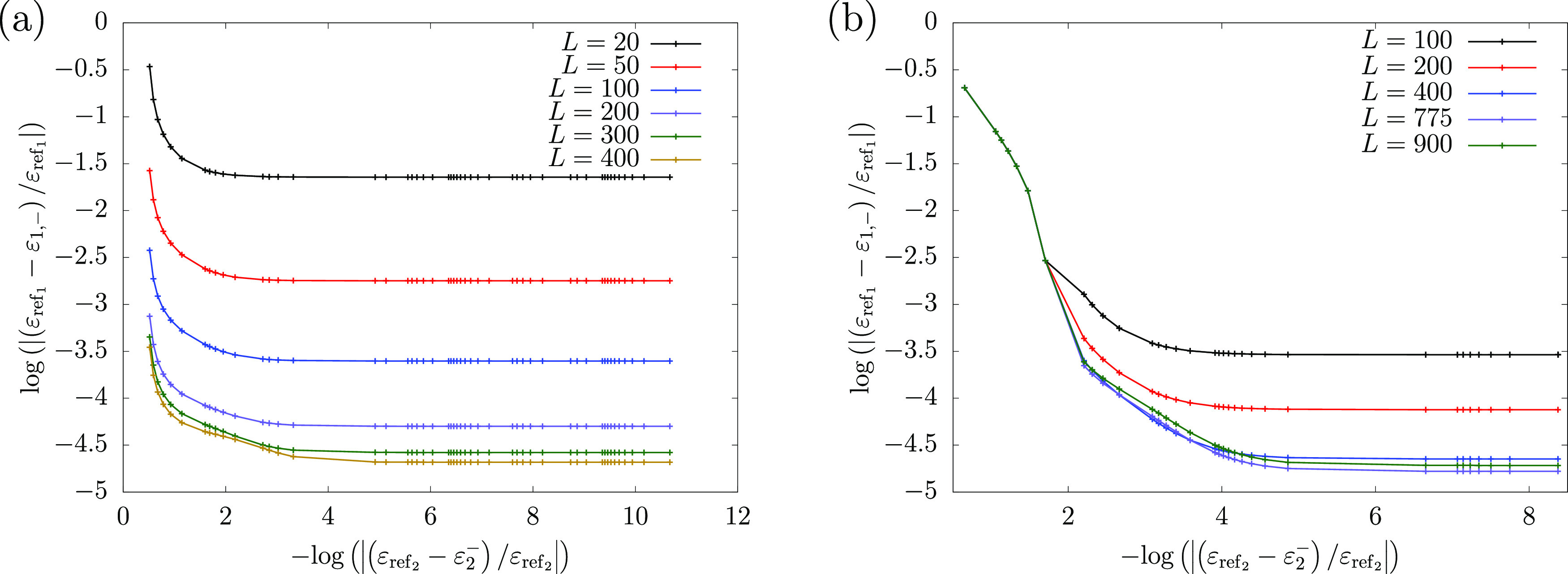
Relative error in the ground state PM lower bound energy as a function of the relative error in ε_2_^–^ for (a) helium and (b) lithium. The ε_ref_*n*__ reference ground- (*n* = 1) and first excited- (*n* = 2) state energies for both atoms are taken from refs (65 and 66), respectively. Regarding the theoretical background of the observed stability of the PM energy, see the Appendix of ref (41).

Unless stated otherwise, PM lower bounds for the ground state energy are computed using the lowest excited state eigenenergy; that is, we replace *x*_1_(ε_1_) with ε_2_^–^ (see Table 2) in the PM equation to generate the lower bound ε_1,–_. (The minus sign used as a superscript denotes an estimated value, in contrast to the subscript notation which indicates a “true” lower bound.) In practice, one usually would not know the first excited state energy exactly without knowing the ground state one with a similar or higher accuracy. This is not a key issue, though, since the lower bound estimate (in either Temple, Lehmann, or PM theory) is rather insensitive to the precise value one uses for the excited state energy. This is shown for the PM lower bound in Figure 2 where we plot the accuracy of the lower bound for the ground state energy of He (Figure 2a) and Li (Figure 2b) as a function of the accuracy of the “pole” energy (ε_2_^–^). For example, when the relative error of the pole energy for He and Li is 10^–2^, the relative error of the PM energy is ca. 2 orders of magnitude lower, 10^–4^ (with at least *L* = 200 and 400 basis function for He and Li, respectively). The common adopted strategy in lower bound calculations is to use the largest known value of the “pole”—which provides the best lower bound estimate—to the eigenvalues whose energy is lower than the “pole” energy.

**Table 2:** Excited State Energies, in E_h_^a^

upper bounds (literature data):				
	ε_2,+_	ε_3,+_	ε_4,+_	ε_5,+_
He^65^	–2.145 974 046 05	–2.061 271 989 7	–2.033 586 717 0	–2.021 176 851 57
Li^66^	–7.354 098 369	–7.318 530 665	–7.303 551 5	–7.295 859

lower bounds (estimate):				
	ε_2_^–^	ε_3_^–^	ε_4_^–^	ε_5_^–^
He	–2.145 974 046 10	–2.061 271 989 8	–2.033 586 717 1	–2.021 176 851 60
Li	–7.354 098 4	–7.318 54	–7.303 552	–7.295 9

a The ε_*n*,+_ upper bound values are taken from the literature and ε_*n*_^–^ estimated lower bound values were created as ε_*n*_^–^ = ε_*n*,+_ – δ. For helium, δ ≈ 10^–10^ E_h_. For lithium, δ is between 10^–7^ and 10^–4^ E_h_, depending on the state. The computed lower-bound energies reported in this work were found to be relatively insensitive to (orders of magnitude) changes in δ (see Fig. 2 and the appendix of ref (41)).

### On the Convergence of Singular Expectation Values When Using a Gaussian Basis Set

In earlier work, it was found that the PM lower bound had a convergence rate comparable to the Ritz upper bound with respect to the basis set size.^41^ In the present work using ECGs, when the basis set size is small, the convergence curves for the Ritz upper bound and the PM lower bound run almost parallel to each other (Figure 1). As the ECG basis set is increased, we observe (Figure 1b–d) an unexpected slowdown in the convergence rate of the PM energy for He, Li^+^, and Li that starts at around a 10 ppb relative precision of the Ritz energy, leading to a difficulty in converging the PM bound to a relative precision better than 10 ppm. This behavior may be connected with the missing electron–nucleus cusp in the spatial basis function.

**Figure 3:**
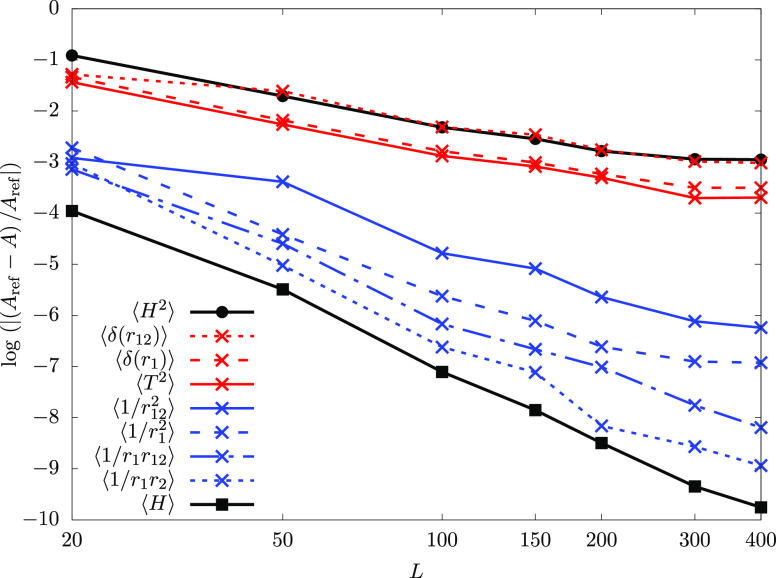
Convergence of the expectation values of *H*, *H*^2^, and contributing terms to *H*^2^ computed with the ground-state (Ritz) eigenfunction of the helium atom. The *A*_ref_ reference values are taken from refs (65 and 68).

To better understand the origin of the slowdown in the PM bound convergence rate, we analyzed the convergence properties of ⟨*H*^2^⟩ in comparison with the ⟨*H*⟩ expectation value as obtained with the (Ritz) ground-state eigenfunction (Figure 3). For this analysis, we focus on the two-electron systems, for which the Hamiltonian (in atomic units, with a *Z* nuclear charge number)
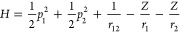
4.1is “well-behaved,” but when it is squared,
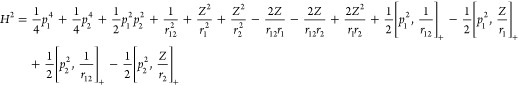
4.2∇^4^- and Δ_***r***_1/*r* = −4*πδ*(***r***)-type “singular” operators appear. These operators appear also in the Foldy–Wouthuysen relativistic perturbation theory^58^ and are known to have nonfavorable convergence properties when using a Gaussian basis due to the lack of a proper representation of the wave function cusps by Gaussian functions.^52,53^Figure 3 shows the convergence behavior for the terms in *H*^2^ and indeed shows that the “singular” operators are responsible for the slowdown in the convergence of the ⟨*H*^2^⟩ expectation value calculated with the ground-state Ritz eigenvector. In principle, it should be possible to use (and extend) the integral transformation (IT) technique^52,53^ to include the analytic short-range behavior near the cusps of the exact wave function during the evaluation of ⟨*H*^2^⟩. Generalization of the IT technique should be straightforward to 1/*r*^2^-type terms along the lines of ref (53), but some further considerations may be necessary for the IT evaluation of -type “mixed” kinetic-Coulomb terms.

### Variance Minimization

For (at least partially) overcoming the slow convergence of ⟨*H*^2^⟩ with the finite basis set size, we have reconsidered the selection procedure of both the nonlinear as well as the linear parameters in our approximate wave function. In the initial applications (Figure 1), the (finite-dimensional) basis set was generated and refined based on the (ground-state) energy minimization condition. For a better representation of the *H*^2^ operator on our finite dimensional subspace, we have replaced the energy-minimization condition with the (ground-state) variance-minimization condition for the selection and refinement of the ECG basis functions.

**Figure 4:**
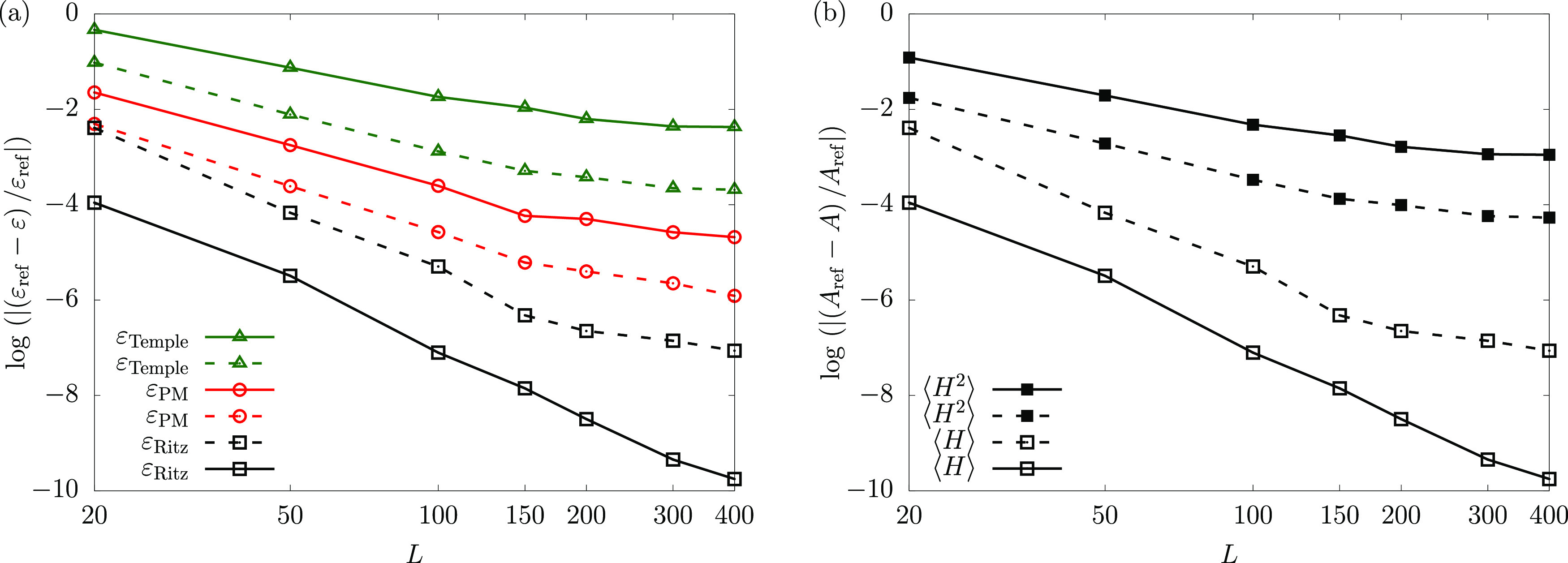
Convergence plots for (a) the upper and lower bound energies of the helium ground state; (b) the ⟨*H*^2^⟩ and ⟨*H*⟩ expectation values for the helium ground state. Solid lines represent energy-optimized basis sets and dashed lines represent variance-optimized basis sets.

First, we tested this “variance-optimization” approach for the ground state of helium (Figure 4). The figure shows that the selection of (the nonlinear parameters of) the ECG functions by minimization of the ground-state variance mitigates the effect of the singular operators in the *H*^2^ operator (and thereby, the corresponding regions of the wave function closer to the cusps are more accurate) during the basis selection, and thus (by construction) a faster convergence of the ⟨*H*^2^⟩ expectation values is observed (Figure 4b). We also obtain lower variances, and thus tighter lower bounds overall. Most importantly, we reach the subppm range for the relative precision of the lower-bound (PM) ground-state energy for helium (Figure 4a).

Despite the improved performance of this approach for the helium ground state, its direct application to the lithium atom was not successful, due especially to the small gap between the first and second excited state energies.

### Lehmann-Generated Basis Vectors

The matrices of the Hamiltonian and Hamiltonian squared operators over a finite-dimensional subspace do not commute and thus, cannot be simultaneously diagonalized. Nevertheless, we would like to have a basis for our finite-dimensional subspace (that is used to implement the computations), for which the two matrices are near-simultaneously diagonalized, or in other words, we would like to have a “good” basis that is “in between” (in some, well-defined sense) the Ritz eigenvectors and the variance eigenvectors.

A meaningful choice within the context of lower bound theory is provided by the solutions of the Lehmann equation, eq 2.21.^18,19,69^ To achieve this goal, we constructed a not necessarily orthonormal basis, using the (normalized) Lehmann eigenfunctions |Ω_*j*_^(*L*)^⟩, *j* = 1, ···, *L*. With these functions we computed the diagonal matrix elements (expectation values) of the Hamiltonian and their associated variances, eqs 2.31 and 2.32. Then, we (numerically) checked, as discussed in the PM theory section and presented in the Supporting Information, that the condition *x*_1_(ε_1_) ≥ ε_2_ was fulfilled. In the following sections, we will refer to this procedure as the Lehmann-based PM lower bound theory. We also note that we will refer to the Temple lower bound obtained with the ground state Lehmann eigenfunction as the Lehmann lower bound to the ground-state energy.^18,19,69^

**Figure 5:**
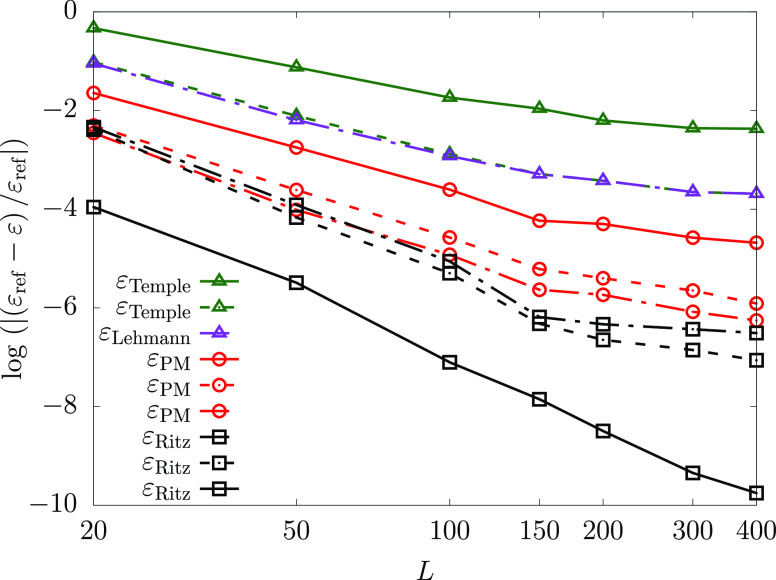
Convergence of the Ritz (□) upper and the Temple (△) and PM (○) lower bounds for the example of the helium atom ground-state energy using energy-optimized (solid), variance-optimized (dashed) basis functions, and Lehmann eigenvectors (dashed/dotted). We note that the Temple bound (△) obtained with the Lehmann eigenvectors (purple, dashed/dotted) is, by virtue of eq 2.25, identical to the Lehmann lower bound and we refer to it as the Lehmann bound.

In our computations for helium, the Lehmann pole ρ was set to an estimated lower bound to the first excited state, ε_2_^–^ (Table 2 presents the collection of the excited-state energies for helium and lithium: tight upper bounds taken from the literature and the estimated lower bounds which are constructed and used in this work). For the helium ground-state energy, the Temple bound over a variance-optimized subspace was already good, and the selection of the Lehmann eigenvectors as a basis over this subspace resulted only in a minor improvement for the lower-bound energy (Figure 5). At the same time, switching to the Lehmann basis instead of the Ritz (eigenvector) basis resulted in an order of magnitude improvement for the PM energy (Figure 5). This improvement was observed in addition to the approximately 1 order of magnitude improvement of the PM bound obtained by using the (ground-state) variance-optimized selection of the subspace instead of the energy optimization procedure (Figure 4).

This procedure of choosing a Lehmann generated basis for a ground-state, variance-optimized subspace that led to 2 orders of magnitude improvement for the helium ground-state PM energy, did not however turn out to be as useful for improving the lithium ground-state PM bound.

We have further experimented with particular selections and combinations of the various parameters in the theory. PM theory allows for an “internal” check to ensure that one really obtains a lower bound to the exact energy (in our case, the ground-state energy). As explained when presenting the PM method in the second section of this paper, following eqs 2.29 and 2.30, one has to ensure that *x*_1_(ε_1_) ≥ ε_2_. For this purpose, one has to study the behavior of *x*_1_ as a function of the basis set at a fixed value of ε, which is in the vicinity of the ground-state energy. In addition, one has to have a lower-bound estimate for the excited-state energy. Since for all systems studied in this work, high-quality upper bounds are available in the literature for the excited state energies, we could “create” a “rough” lower-bound estimate to the excited state energy. For the particular choices of the parameters in the theory, the tests taken to ensure that *x*_1_(ε_1_) ≥ ε_2_ are described and provided in the Supporting Information.

**Figure 6:**
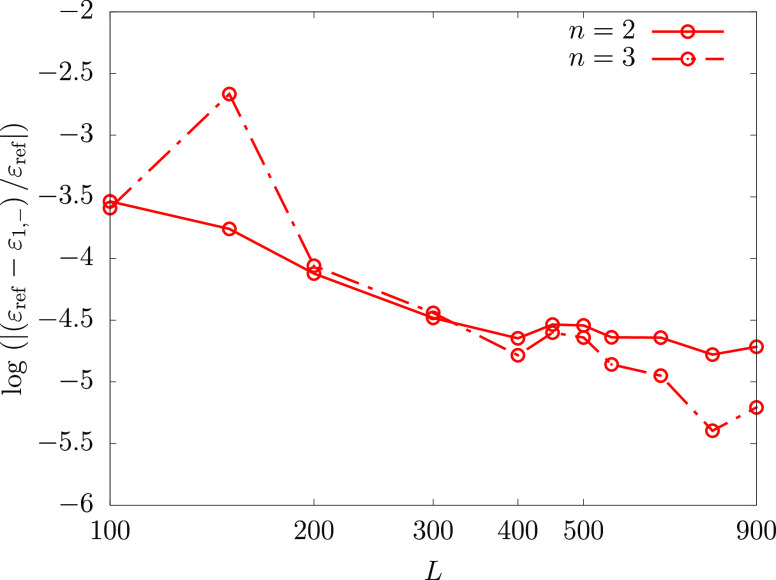
PM lower bounds for different excited state energies ε_*n*_^–^ used in the PM equation for the Li atom. The solid line is the PM bound shown in Figure 1d and the dashed line is the PM lower bound calculated using ε_3_^–^ and a Lehmann generated basis set. (For the ε_*n*_^–^ values see Table 2.).

We have experimented with the Lehmann pole ρ, eq 2.21, and the energy parameter ε, which is the input to the PM equation, eq 2.30, and set them equal to the (estimated) relevant lower bounds for higher excited states (see Table 2). For helium, this approach did not lead to any significant improvement for the Temple bound. However, when this parameter was set equal to a lower bound for the second-excited state of lithium, combined with using the Lehmann generated basis set, a significant improvement in the PM lower bound accuracy was observed. This can be seen in Figure 6, which shows that using ε_3_^–^ (Table 2) in the computations, for both ρ and ε, and with a basis set of dimension *L* = 775, we obtained a PM lower bound for the ground state energy of −7.478 090 346 E_h_ that is accurate to 3 × 10^–5^ E_h_, equivalent to a relative precision of 4 ppm; this represents the most accurate lower bound to the lithium ground state to date.

**Figure 7:**
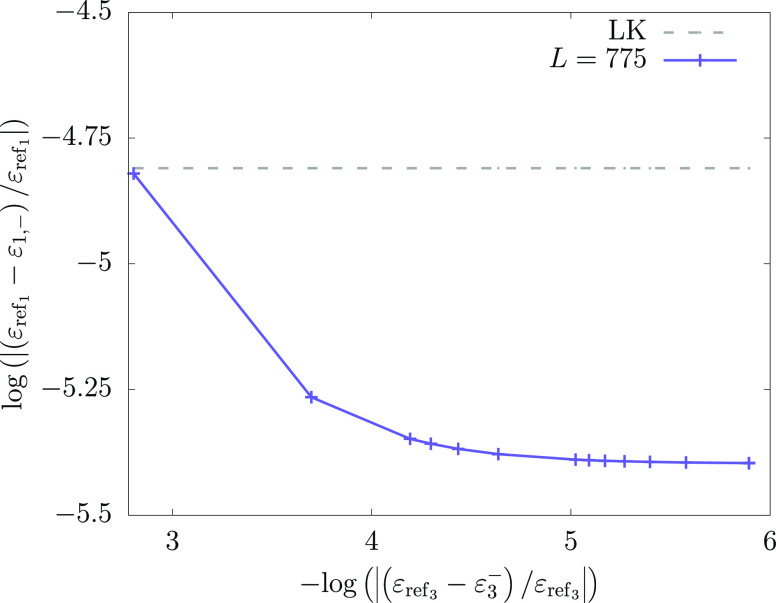
Insensitivity of the best PM lower bound energy computed in this work for lithium with respect to the ε_3_^–^ pole energy used in the Lehmann–PM computations. The best lower-bound value available from literature^21^ is also shown (dashed line). The ε_ref_*n*__ reference ground- (*n* = 1) and second excited- (*n* = 3) state energies are taken from ref (66).

Here too, the accuracy of the lower bound is rather insensitive to the precise value of ε_3_^–^. This may be seen in Figure 7 where the (log of the) relative accuracy (solid line) of the PM lower bound to the ground state energy of Li is plotted vs the accuracy of the lower bound used for ε_3_^–^ and compared with the accuracy of the Lüchow–Kleindienst lower bound (horizontal dashed line) to the ground state energy. The validity of the Lehmann lower bound requires that ε_3_^–^ is larger than λ_2_ and this limits the values used in the figure.

**Table 3:** Energy Bounds for the Helium Ground State Obtained with Energy (E)- and Variance (V)-Optimized Basis Sets Using *L* = 400 ECG Functions^a^

energy bound	optimization	basis	ε_1_ [E_h_]
Ritz	E	R	–2.903 724 376 5
ref (35)., Ritz (41 digits)			–2.903 724 377 0 ...
	E	R	–2.916 136 676
Temple	V	R	–2.904 321 557
Lehmann (ε_2_^–^)	V	L (ε_2_^–^)	–2.904 320 052
	E	R	–2.903 784 830
PM (ε_2_^–^)	V	R	–2.903 727 924
	V	L (ε_2_^–^)	–2.903 725 983
ref (35)., Temple (32 digits)			–2.903 724 377 ...

a Either Ritz (R) or Lehmann (L) basis vectors are used for the computation of the lower bounds. The Lehmann pole and the PM parameter is indicated in parentheses (for the ε_*n*_^–^ values see Table 2).

**Table 4:** Energy Bounds for the Lithium Ground State Obtained with *L* ECGs Optimized to the Ground-State Energy (E)^a^

energy bound	optimization	basis	ε_1_ [E_h_]
Ritz (*L* = 900)	E		–7.478 060 309
ref (67)., Ritz (Hyll., *L* = 9577, 14 digits)			–7.478 060 323 892 4
Temple (*L* = 900)	E	R	–8.766 772 761
Weinstein (*L* = 900)	E	L(ε_3_^–^)	–7.875 888 335
Lehmann(ε_3_^–^) (*L* = 900)	E	L(ε_3_^–^)	–8.471 368 155
PM(ε_2_^–^) (*L* = 775)	E	R	–7.478 184 715
PM(ε_3_^–^) (*L* = 775)	E	L(ε_3_^–^)	–7.478 090 347
ref (21)., Lehmann (Hyll., *L* = 920, 4 digits)			–7.478 176

a Either Ritz (R) or Lehmann (L) basis vectors are used for the computation of the lower bounds. The Lehmann pole and the PM parameter is indicated in parentheses (for the ε_*n*_^–^ values see Table 2).

### Toward Systematic Improvement of the PM Lower Bound Using ECGs

Figure 1d shows that increasing the dimensionality of the subspace (the number of ECG basis functions) does not necessarily ensure an improvement of the PM lower bound, in contrast to the Ritz energy, which improves monotonically upon the increase in the basis set size.

**Figure 8:**
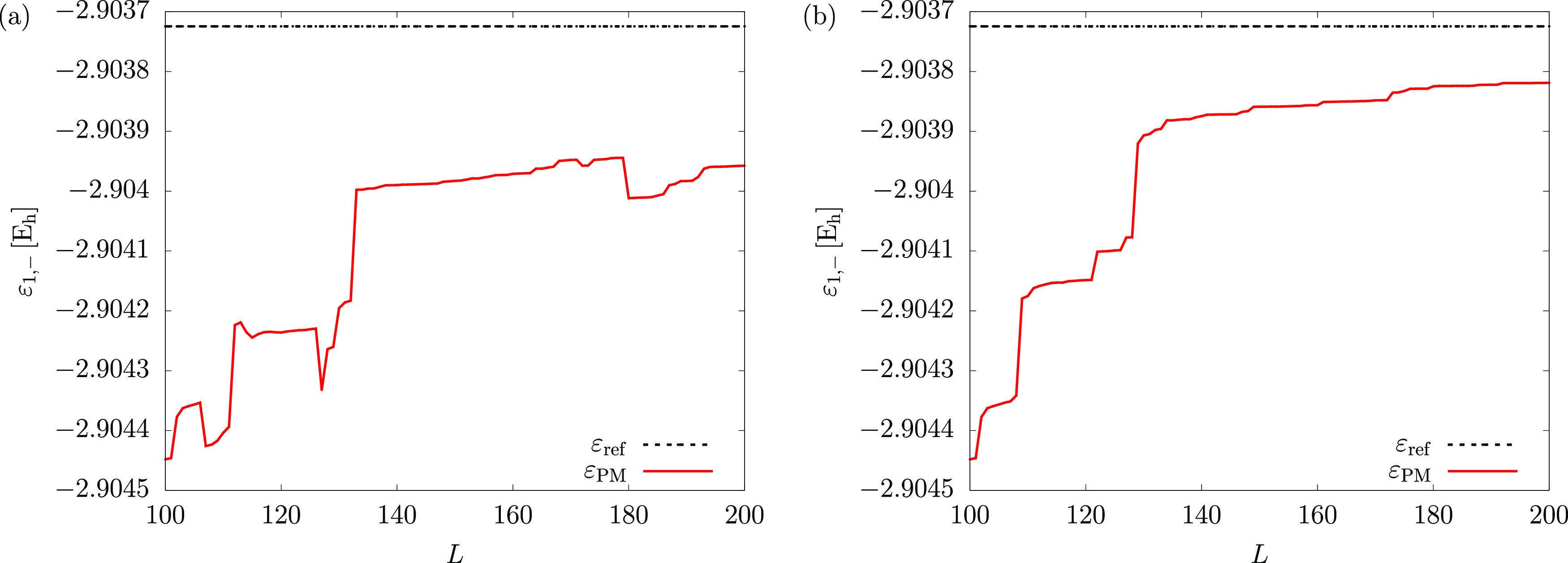
Monotonicity check for the PM lower bound to the ground-state energy of the helium atom upon enlargement of the basis set. Basis functions selected based on (a) solely the Ritz energy-minimization condition or (b) a combination of the Ritz energy-minimization condition and the PM monotonicity check. The reference energy (upper dotted line) is taken from ref (35). (for which the first 11 digits are reproduced in Table 3).

This observation led us to carry out a simple test calculation regarding the monotonicity of the PM lower bound. This test was initiated for the helium atom with 100 ECG functions tightly optimized using the (ground-state) energy minimization condition. New ECG functions were then added one-by-one using the stochastic variational method but without further refinement, and most importantly, the original 100 ECG functions were kept fixed (no refinement either). A single ECG was first added to this set, and the PM lower bound was calculated for this new basis set (of 101 functions), after which another ECG was added. This procedure was repeated until 200 basis functions were present in the set. Figure 8a shows that the PM lower bound is indeed not a monotonically increasing function with respect to the number of (ground-state) energy-optimized basis functions. This test calculation highlights the origin of the increase in the PM lower bound values for the lithium ground state around 400–550 basis functions, and again for 900 basis functions (Figure 1d).

The lack of monotonicity of the PM lower bound with increasing basis set size is due to the fact that the current basis function generation (and also refinement) routine selects basis functions solely based on the energy-minimization condition, which does not necessarily improve the PM lower bound (Figure 8a). Inspection of the PM equation, eq 2.29, suggests that other quantities, such as the ground-state variance and excited-state energies and variances, influence the value of the bound. For a systematic, monotonic improvement of the PM bound, some further conditions are needed for the basis generation and its refinement.

For the practical realization of such a procedure, in addition to the minimization of the energy, we need to find a way to ensure that the PM lower bound does not decrease during the basis function generation (refinement) procedure. We have implemented this idea, by directly monitoring the PM bound: the “new” acceptance criterion for a new basis function is that it must reduce the energy by the largest amount (within a trial set) *and* that it must not lower the PM bound. This procedure, by construction, ensures a monotonic improvement of both the Ritz upper bound and the PM lower bound (Figure 8b). During the computations shown in the figure, we had to discard in the “worst case” the first 26 “best” functions (out of 1000 trial functions ordered according to the new Ritz energies) and select the 27th best for the basis set to ensure monotonicity of the PM lower bound to the energy. It is evident from Figure 8b that this algorithm goes a long way in improving the quality of the PM lower bound.

This extra condition does somewhat increase the computational expense of the procedure, since we currently do not have any fast PM update algorithm (unlike the fast rank-1 Ritz eigenvalue update procedure^45,46^). A similar additional condition could be included also in the basis refinement process, but the computational cost would increase simultaneously. For further developments, a better understanding of the interplay of the ground- and excited-state properties affecting the PM lower bound will help to develop an efficient and systematic basis generation and selection procedure.

## Summary and Conclusions

The Ritz method is well-known to give accurate upper bounds to exact eigenvalues, and its application in quantum chemistry and physics is widespread. However, an equivalent method for calculating lower bounds of similar quality is still in its infancy.

In this paper, we applied the recently formulated Pollak–Martinazzo lower bound theory to some nontrivial atomic systems. Using ECG basis sets, we have demonstrated that one can obtain lower bounds to the ground-state energy with a precision of parts per million or better. The ground state of the helium atom was used as a primary test system. Three different approaches were investigated for selecting the basis functions: energy minimization, variance minimization, and the use of Lehmann eigenvectors for a selected subspace to build the Hamiltonian and Hamiltonian squared expectation values. In all examples, the PM lower bound theory consistently returned the best lower-bound energies, orders of magnitude better than the Temple and Weinstein bounds. All three approaches lead to increasingly more-accurate PM lower bounds for the helium ground-state energy.

Why is the PM method so much more accurate than Lehmann’s theory? After all, both use as input the Ritz eigenvalues and matrix elements of the *H*^2^ matrix. One should note that Lehmann’s theory is at the end of the day, as also discussed in the second section of this paper, an optimized Temple lower bound. As is also shown, any Temple lower bound is derived through a Cauchy–Schwartz inequality implying that, for a given finite basis set (which is not the exact basis set), the Lehmann lower bound cannot be exact. This is not the case for the PM lower bound. Consider for example the ground-state energy. If *x*_1_(ε_1_) is known exactly, then one may solve for the exact ground-state energy. The quality of the lower bound depends on how close our lower bound estimate of *x*_1_ is to the exact *x*_1_(ε_1_). As the basis set is increased, the lower bound estimate to *x*_1_ comes closer to the exact value so that the lower bound is rather accurate, and in any case, much more accurate than the Lehmann–Temple optimal lower bound.

Our best lower bound value for the ground-state energy of helium, of 0.3 ppm relative precision, was obtained with the PM theory using Lehmann eigenvectors for a ground-state variance-optimized subspace. Within this setup (basis set, optimization procedure, CPU usage) the 3:10^7^ relative precision of the lower-bound energy should be compared with the 1:10^9^ relative precision of the upper bound energy. This more-than 2 orders of magnitude difference of PM theory does challenge us to further improve the ECG-based lower bound computation.

To obtain ppm accuracy also for the Li atom, the PM lower bound theory was generalized and formulated to enable to incorporate in it diagonal matrix elements and associated variances, valid even when the basis set is not necessarily orthogonal. The disadvantage of this generalization is that the diagonal elements of the Hamiltonian do not necessarily interleave with the exact eigenvalues, so that it is more difficult to create an objective criterion for the validity of the assumption that *x*_*j*_(ε_1_) ≥ ε_*j*+1_. To overcome this difficulty, we had to use known results for the various eigenvalues, to ensure the property. More work is needed to provide an objective criterion for the validity of the condition. One possibility is by studying the analytic properties of solutions of the PM equation, and such work is underway. We stress that this generalization and the use of the Lehmann nonorthogonal basis set was essential to obtain a lower bound with ppm accuracy.

In the case of a lower bound to the ground state of Li, an energy-optimized subspace, combined with the Lehmann-generated basis returned an improved PM lower bound for the lithium ground state when the Lehmann pole was set equal to a lower bound to the second-excited state of lithium. The relative precision of this lower bound is 4 ppm (the absolute precision is 3 × 10^–5^ E_h_). This represents an improvement of a factor of almost 4 upon the earlier best (Lehmann) bound of 15 ppm precision.^21^

These results indicate the potential of the PM lower bound theory coupled with the employment of ECGs. This would not have been possible without the recently developed analytic theory for computation of *H*^2^ matrix elements. It was also emphasized in the paper that we are far from exhausting further routes for systematic improvements to the basis set optimization procedure. Most importantly, the energy minimization combined with a PM check to ensure that the PM lower bound increases monotonically as the basis set is increased appears to be a promising route.

All computations presented in this work rely on the explicit calculation of variances. This increases the computational expense involved in the practical application of the Pollak–Martinazzo theory, for example, in quantum chemistry. An extrapolation procedure based on eq 2.9 that would obviate the need for an explicit calculation of the *H*^2^ matrix was recently explored,^42^ and initial results for the hydrogen atom using an odd Gauss–Hermite basis are promising. Further work is necessary to test this strategy for Gaussian-type basis functions, the mathematical properties of which differ from those of orthogonal polynomials.

This work was dedicated to the practical implementation of PM lower bound theory for few-electron atomic systems at the most basic level of an already meaningful theoretical description. In principle, the methodology described in this work can be adapted also for the computation of electronic energies of molecules within and even without the Born–Oppenheimer approximation.^14,46^ This will require the replacement of the plain ECG functions, eq 3.2, with “floating” ECGs and the corresponding generalization of the newly implemented 1/*r*_*ij*_*r*_*kl*_ integral expressions (see ref (47) and the Supporting Information). It would be of significance to complement the five-particle upper bound^70,71^ with a similarly precise lower bound for selected rovibronic states of the H_3_^+^ molecular ion, for which a 10 ppm relative precision would allow for the assessment of the importance of nonadiabatic (in comparison with Born–Oppenheimer results and corrections to them) and relativistic quantum electrodynamics (in comparison with experiment) “effects.”

## Supporting Information Available

Derivation of matrix elements; fulfillment of the *x*_*j*_(ε_1_) ≥ ε_*j*+1_ condition (pdf)
