# Supplementary material for: Lower Bounds for Nonrelativistic Atomic Energies
Source: ACS Phys Chem Au. 2021 Sep 20;2(1):23–37. doi: 10.1021/acsphyschemau.1c00018 (PMC8796283; doi:10.1021/acsphyschemau.1c00018)
Supplement: Supplementary file 1 — pg1c00018_si_002.pdf [file pg1c00018_si_002.pdf]

Lower bounds for non-relativistic atomic energies

Robbie T. Ireland,[†] Peter Jeszenszki and Edit Mátyus ,[*,‡] Rocco Martinazzo,[*,¶] and Miklos Ronto and Eli Pollak[*,§]

†*Institute of Chemistry, Eötvös Loránd University, Pázmány Péter sétány 1/A, Budapest, H-1117, Hungary,*

and School of Chemistry, University of Glasgow, University Avenue, G12 8QQ, Glasgow, United Kingdom

‡*Institute of Chemistry, Eötvös Loránd University, 1117 Budapest, Hungary*

¶*Department of Chemistry, University of Milan, Milan,*

and Institute of Molecular Science and Technologies (ISTM), Consiglio Nazionale delle Ricerche (CNR), Milan, Italy

§*Chemical and Biological Physics Department, Weizmann Institute of Science, 76100, Rehovot, Israel*

E-mail: edit.matyus@ttk.elte.hu; rocco.martinazzo@unimi.it; eli.pollak@weizmann.ac.il

Supporting Information

Derivation of Matrix Elements

The $\left\langle \frac{1}{r_{ij}r_{pa}} \right\rangle$ expectation value, corresponding to the product of the electron–electron and electron–nuclei potential energy operators, can be solved by beginning with Eq. 3.1,

$$\psi(\boldsymbol{r}, \boldsymbol{\sigma}) = \mathcal{A}\{\phi(\boldsymbol{r}, \boldsymbol{A})\chi(\boldsymbol{\sigma}, \theta)\} \ . \tag{S1}$$

The spin functions are constructed by coupling elementary spin functions to the selected total spin quantum numbers. As such, it is the spatial ECG functions $\phi(\boldsymbol{r}, \boldsymbol{A})$ that are of interest when deriving the expectation value, where the kth ECG basis function is written as

$$\phi_k(\boldsymbol{r}, \boldsymbol{A}) = \exp\left[-\boldsymbol{r}^T \left(\boldsymbol{A}_k \otimes \boldsymbol{I}_3\right) \boldsymbol{r}\right] \ . \tag{S2}$$

$\boldsymbol{r} \in \mathbb{R}^{3n_\mathrm{p}}$ is the vector containing the Cartesian positions of the n_p particles, $\boldsymbol{A}_k \in \mathbb{R}^{n_\mathrm{p} \times n_\mathrm{p}}$ is the positive-definite symmetric matrix containing the non-linear parameters of the kth ECG basis function and $\boldsymbol{I}_3 \in \mathbb{R}^{3\times 3}$ is the identity matrix. Then, the kth, lth element takes the form

$$\langle\phi_k|\frac{1}{r_{ij}r_{pa}}|\phi_l\rangle = \int_{-\infty}^{+\infty} \mathrm{d}\boldsymbol{r} \ \frac{1}{r_{ij}r_{pa}} \exp\left[-\boldsymbol{r}^T \underline{\boldsymbol{A}}_{kl}\boldsymbol{r}\right] \ , \tag{S3}$$

where we have defined $\underline{\boldsymbol{A}}_{kl} = (\boldsymbol{A}_k \otimes \boldsymbol{I}_3) + (\boldsymbol{A}_l \otimes \boldsymbol{I}_3)$. By using the following Gaussian integral transforms,

$$\frac{1}{r_{ij}} = \frac{2}{\sqrt{\pi}} \int_0^{+\infty} \mathrm{d}t \ \exp\left[-\boldsymbol{r}^T \underline{\boldsymbol{J}}_{ij}\boldsymbol{r}t^2\right] , \tag{S4}$$

$$\frac{1}{r_{pa}} = \frac{2}{\sqrt{\pi}} \int_0^{+\infty} \mathrm{d}u \, \exp\bigl[-\boldsymbol{r}^T \underline{\boldsymbol{J}}_{pa} \boldsymbol{r} u^2\bigr] \ , \tag{S5}$$

we arrive at

$$\langle \phi_k | \frac{1}{r_{ij} r_{pa}} | \phi_l \rangle = \frac{4}{\pi} \int_0^{+\infty} \mathrm{d}t \int_0^{+\infty} \mathrm{d}u \int_{-\infty}^{+\infty} \mathrm{d}\boldsymbol{r} \, \exp\bigl[-\boldsymbol{r}^T (\underline{\boldsymbol{A}}_{kl} + t^2 \underline{\boldsymbol{J}}_{ij} + u^2 \underline{\boldsymbol{J}}_{pa}) \boldsymbol{r}\bigr] \,, \tag{S6}$$

where $\boldsymbol{J}_{mn} \in \mathbb{R}^{n_\mathrm{p} \times n_\mathrm{p}}$ is a rank-1 matrix with element

$$(\boldsymbol{J}_{mn})_{\alpha\beta} = \delta_{m\alpha}\delta_{m\beta} + \delta_{n\alpha}\delta_{n\beta} - \delta_{m\alpha}\delta_{n\beta} - \delta_{n\alpha}\delta_{m\beta} \ . \tag{S7}$$

Furthermore, we define $\underline{\boldsymbol{J}}_{mn} = \boldsymbol{J}_{mn} \otimes \boldsymbol{I}_3$. Using the Gaussian integral expression

$$\int_{-\infty}^{+\infty} \mathrm{d}\boldsymbol{r} \, \exp\bigl[-\boldsymbol{r}^T \boldsymbol{M} \boldsymbol{r}\bigr] = \frac{\pi^{\frac{3n}{2}}}{|\boldsymbol{M}^{\frac{1}{2}}|} \ , \tag{S8}$$

for n-electrons, we obtain

$$\langle \phi_k | \frac{1}{r_{ij} r_{pa}} | \phi_l \rangle = \frac{4}{\pi} \pi^{\frac{3n}{2}} \int_0^{+\infty} \mathrm{d}t \int_0^{+\infty} \mathrm{d}u \, |\boldsymbol{A}_{kl} + t^2 \boldsymbol{J}_{ij} + u^2 \boldsymbol{J}_{pa}|^{-\frac{3}{2}} \ . \tag{S9}$$

The determinant above can be rewritten an

$$|\boldsymbol{A}_{kl} + t^2 \boldsymbol{J}_{ij} + u^2 \boldsymbol{J}_{pa}|^{-\frac{3}{2}} = |\boldsymbol{I} + t^2 \boldsymbol{J}_{ij} \boldsymbol{A}_{kl}^{-1} + u^2 \boldsymbol{J}_{pa} \boldsymbol{A}_{kl}^{-1}|^{-\frac{3}{2}} |\boldsymbol{A}_{kl}|^{-\frac{3}{2}} \ , \tag{S10}$$

meaning we can use the following rank-2 determinant expression,

$$\begin{aligned} & |\boldsymbol{G} + \boldsymbol{H}_1 + \boldsymbol{H}_2| \\ = \ & |\boldsymbol{G}| \left[1 + \mathrm{Tr}\bigl\{\boldsymbol{H}_1 \boldsymbol{G}^{-1}\bigr\} + \mathrm{Tr}\bigl\{\boldsymbol{H}_2 \boldsymbol{G}^{-1}\bigr\} + \mathrm{Tr}\bigl\{\boldsymbol{H}_1 \boldsymbol{G}^{-1}\bigr\} \mathrm{Tr}\bigl\{\boldsymbol{H}_2 \boldsymbol{G}^{-1}\bigr\} - \mathrm{Tr}\bigl\{\boldsymbol{H}_1 \boldsymbol{G}^{-1} \boldsymbol{H}_2 \boldsymbol{G}^{-1}\bigr\}\right] \ , \end{aligned}$$

where $\mathrm{rank}(\boldsymbol{H}_1) = \mathrm{rank}(\boldsymbol{H}_2) = 1$.

For matrices $\boldsymbol{A}$ and $\boldsymbol{B}$, it can be shown that rank $(\boldsymbol{AB}) = $ rank $(\boldsymbol{A})$, if $\boldsymbol{B}$ is a non-singular matrix. As such, rank $\left(\boldsymbol{J}_{ij}\boldsymbol{A}_{kl}^{-1}\right) = $ rank $(\boldsymbol{J}_{ij})$. As each column of $\boldsymbol{J}_{ij}$ is a linear combination of the first, it is a rank-1 matrix, meaning that the $\boldsymbol{J}_{ij}\boldsymbol{A}_{kl}^{-1}$ matrix is also rank-1. The same is true for $\boldsymbol{J}_{pa}\boldsymbol{A}_{kl}^{-1}$.

By letting $\boldsymbol{H}_1 = t^2\boldsymbol{J}_{ij}\boldsymbol{A}_{kl}^{-1}$ and $\boldsymbol{H}_2 = u^2\boldsymbol{J}_{pa}\boldsymbol{A}_{kl}^{-1}$, the expectation value then reads

$$\langle\phi_k|\frac{1}{r_{ij}r_{pa}}|\phi_l\rangle = \frac{4}{\pi}\pi^{\frac{3n}{2}}|\boldsymbol{A}_{kl}|^{-\frac{3}{2}}\int_0^{+\infty}\mathrm{d}t\int_0^{+\infty}\mathrm{d}u\ \left[1+t^2a+u^2b+u^2t^2(ab-c)\right]^{-\frac{3}{2}}\ , \tag{S11}$$

where we have defined

$$a = \mathrm{Tr}\left\{\boldsymbol{J}_{ij}\boldsymbol{A}_{kl}^{-1}\right\}\ , \tag{S12}$$

$$b = \mathrm{Tr}\left\{\boldsymbol{J}_{pa}\boldsymbol{A}_{kl}^{-1}\right\}\ , \tag{S13}$$

$$c = \mathrm{Tr}\left\{\boldsymbol{J}_{ij}\boldsymbol{A}_{kl}^{-1}\boldsymbol{J}_{pa}\boldsymbol{A}_{kl}^{-1}\right\}\ . \tag{S14}$$

We can then see that

$$\begin{aligned}1+t^2a+u^2b+u^2t^2(ab-c) &= 1+t^2a+u^2b+u^2bt^2a-u^2t^2c\\ &= (1+t^2a)\left(1+u^2b-u^2\frac{t^2c}{1+t^2a}\right)\\ &= (1+t^2a)\left[1+u^2\left(b-\frac{t^2c}{1+t^2a}\right)\right]\ ,\end{aligned} \tag{S15}$$

which gives

$$\langle\phi_k|\frac{1}{r_{ij}r_{pa}}|\phi_l\rangle = \frac{4}{\pi}\pi^{\frac{3n}{2}}|\boldsymbol{A}_{kl}|^{-\frac{3}{2}}\int_0^{+\infty}\mathrm{d}t\int_0^{+\infty}\mathrm{d}u\,(1+t^2a)^{-\frac{3}{2}}\left[1+u^2\left(b-\frac{t^2c}{1+t^2a}\right)\right]^{-\frac{3}{2}}\ . \tag{S16}$$

The following substitution can then be made,

$$w = \frac{u^2}{1 + u^2\left(b - \frac{t^2 c}{1+t^2 a}\right)} \ , \tag{S17}$$

leading to

$$\begin{aligned}
\langle\phi_k|\frac{1}{r_{ij}r_{pa}}|\phi_l\rangle &= \frac{2}{\pi}\pi^{\frac{3n}{2}}|\boldsymbol{A}_{kl}|^{-\frac{3}{2}}\int_0^{+\infty}\mathrm{d}t\int_0^{\left(b-\frac{t^2c}{1+t^2a}\right)^{-1}}\mathrm{d}w\,(1+t^2a)^{-\frac{3}{2}}\frac{1}{w^{\frac{1}{2}}} \\
&= \frac{4}{\pi}\pi^{\frac{3n}{2}}|\boldsymbol{A}_{kl}|^{-\frac{3}{2}}\int_0^{+\infty}\mathrm{d}t\,(1+t^2a)^{-\frac{3}{2}}\left(b-\frac{t^2c}{1+t^2a}\right)^{-\frac{1}{2}} \ .
\end{aligned} \tag{S18}$$

By making the substitution

$$z = \left(\frac{t^2c}{1+t^2a}\right)^{\frac{1}{2}} \ , \tag{S19}$$

the expectation value then can be written as

$$\langle\phi_k|\frac{1}{r_{ij}r_{pa}}|\phi_l\rangle = \frac{4}{\pi}\frac{\pi^{\frac{3n}{2}}|\boldsymbol{A}_{kl}|^{-\frac{3}{2}}}{\sqrt{c}}\int_0^{\sqrt{\frac{c}{a}}}\mathrm{d}z\,\frac{1}{(b-z^2)^{\frac{1}{2}}} \ . \tag{S20}$$

Finally, we define $z = \sqrt{b}\sin(\rho)$ such that

$$\begin{aligned}
\langle\phi_k|\frac{1}{r_{ij}r_{pa}}|\phi_l\rangle &= \frac{4}{\pi}\frac{\pi^{\frac{3n}{2}}|\boldsymbol{A}_{kl}|^{-\frac{3}{2}}}{\sqrt{c}}\int_0^{\sin^{-1}\left(\sqrt{\frac{c}{ab}}\right)}\mathrm{d}\rho\,\frac{\sqrt{b}\cos(\rho)}{(b-b\sin^2(\rho))^{\frac{1}{2}}} \\
&= \frac{4}{\pi}\frac{\pi^{\frac{3n}{2}}|\boldsymbol{A}_{kl}|^{-\frac{3}{2}}}{\sqrt{c}}\int_0^{\sin^{-1}\left(\sqrt{\frac{c}{ab}}\right)}\mathrm{d}\rho \ .
\end{aligned} \tag{S21}$$

Hence, evaluation of Eq. S21 leads to the final expression

$$\langle\phi_k|\frac{1}{r_{ij}r_{pa}}|\phi_l\rangle = \frac{4}{\pi}\frac{\pi^{\frac{3n}{2}}|\boldsymbol{A}_{kl}|^{-\frac{3}{2}}}{\sqrt{c}}\sin^{-1}\left(\sqrt{\frac{c}{ab}}\right) \ , \tag{S22}$$

for $ab - c > 0$, $c \neq 0$.

This expression can be generalized to the $\left\langle \frac{1}{r_{ij}^2} \right\rangle$ expectation value that appears in the V_{ee}^2 operator of the 2-electron case. Then, the $\underline{\boldsymbol{J}}_{pa}$ matrix can be seen to be equal to the $\underline{\boldsymbol{J}}_{ij}$ matrix. The traces of Eqs. S12–S14 are such that $a = b$ and $c = a^2$, where we have used the fact that for a rank-1 matrix $\boldsymbol{M}$

$$\mathrm{Tr}\left\{\boldsymbol{M}^2\right\} = \left(\mathrm{Tr}\{\boldsymbol{M}\}\right)^2 \ . \tag{S23}$$

Equation S22 then reads as

$$\langle \phi_k | \frac{1}{r_{ij}^2} | \phi_l \rangle = \frac{4}{\pi} \frac{\pi^{\frac{3n}{2}} |\boldsymbol{A}_{kl}|^{-\frac{3}{2}}}{\sqrt{a^2}} \sin^{-1}(1) = \frac{2\pi^{\frac{3n}{2}} |\boldsymbol{A}_{kl}|^{-\frac{3}{2}}}{a} \ . \tag{S24}$$

Fulfillment of the $x_j(\varepsilon_1) \geq \varepsilon_{j+1}$ condition

As discussed in the paper, an essential part of the PM lower bound theory is the assurance of the lower bound property of the poles of the PM equation. For this purpose, we consider two strategies. The first one, used in Ref. 1, is to check whether x_j decreases monotonically when increasing the dimensionality of the basis set. This test is self-contained, but not directly applicable to the current setup with ECGs. This approach is useful if the basis set is increased "systematically" without changing the already selected functions, *e.g.,* as it is in the case of increasing the dimensionality of basis sets composed of orthogonal polynomials. The ECG basis set including functions that are generated and regularly refined based on the energy-minimization condition does not necessarily have this property.

The second strategy is testing the fulfillment of the $x_j(\varepsilon_1) \geq \varepsilon_{j+1}$ condition that requires information about ground and excited-state energies (that are generally not known). In particular, for Li, we know that we have a good upper bound from the ECG computation that can be estimated to be converged on the order of a few ppbs. Then, we may take this

value and lower it by 50 ppb to have a lower-bound estimate. Then, by using this value for ε in the PM equation one finds the pole x_j and checks whether it is greater than ε_{j+1} (that is also taken from some other computation). Is this a "useful" check? In its present form, it requires external information that is, in general, not available. There is work underway, starting out from this particular condition that we check here, to eliminate the need to use information on state energies, if the overall computation is sufficiently accurate.

The results of this (second) test are shown for the Li atom using the energy-optimized basis set in Table S1. The column denoted by $x_1(\varepsilon_1)$ [E$_\mathrm{h}$] shows the value of x_1 found as a function of the dimensionality of the energy-optimized ECG basis set. The last line shows the known value of the first excited state energy. One then notices that for all basis sets used, indeed $x_1 \geq \varepsilon_2$ is fulfilled. Inserting a lower bound to the excited state energy, ε_2^-, in the PM equation gives the lower bound to the ground state energy, shown in the second column. The known converged energies are shown in the last row. The data in this column is also plotted in Fig. 1d of the paper.

Table S1: Li atom ground state energy lower bound: testing the validity of the $x_1(\varepsilon_1) \geq \varepsilon_2$ condition which assures that the PM expression gives a lower bound to the ground state of the Li atom when replacing x_1 with ε_2 in the PM equation. The PM matrix was calculated using the Ritz eigenvectors corresponding to an energy-optimized subspace. (See Table 2 in the paper, for the ε_n values.)

L	$x_0(\varepsilon_2^-)$ [E$_\mathrm{h}$]	λ_2 [E$_\mathrm{h}$]	$x_1(\varepsilon_1)$ [E$_\mathrm{h}$]
50	−7.489 533 004	−7.301 424 115	−7.303 938 484
100	−7.480 233 615	−7.334 043 730	−7.334 299 893
150	−7.479 359 816	−7.340 602 621	−7.340 681 986
200	−7.478 625 005	−7.344 480 432	−7.344 523 855
300	−7.478 306 665	−7.349 351 448	−7.349 359 498
400	−7.478 228 894	−7.351 294 035	−7.351 297 365
450	−7.478 278 264	−7.351 489 282	−7.351 490 824
500	−7.478 274 447	−7.351 593 385	−7.351 594 510
550	−7.478 232 084	−7.351 979 625	−7.351 980 558
650	−7.478 231 080	−7.352 059 482	−7.352 060 145
775	−7.478 184 715	−7.352 827 201	−7.352 827 644
900	−7.478 204 144	−7.352 966 162	−7.352 966 447
ε_ref [2]	−7.478 060 324	−7.354 098 369	

Similar results are shown in Table S2 for the variance-optimized Lehmann basis set results. Here, the fourth column denoted by $x_2(\varepsilon_1)[\mathrm{E_h}]$ is the check whether the second root of the PM equation is greater than or equal to ε_3 for which the precise value is given in the last row of the table. One notes that for all basis set sizes, L, the condition $x_2(\varepsilon_1) \geq \varepsilon_3$ is fulfilled. The last column shows the Lehmann lower bound values that are not accurate in this case.

Table S2: Li atom ground-state energy lower bound: Test for the validity of the condition $x_2(\varepsilon_1) \geq \varepsilon_3$. The test is shown for results based on a variance-optimized Lehmann eigenvector basis set with $\rho = \varepsilon = \varepsilon_3^-$, a lower bound to the second excited state (See Table 2 for the ε_n values used here). Further explanation of the data is provided in the text.

L	$x_1\left(\varepsilon_3^-\right)$ [E$_\mathrm{h}$]	λ_2 [E$_\mathrm{h}$]	$x_2(\varepsilon_1)$ [E$_\mathrm{h}$]	$\tau\left(\varepsilon_3^-\right)$ [E$_\mathrm{h}$]
100	−7.479 975 612	−7.333 411 548	−7.283 362 123	−12.485 954 782
150	−7.494 183 958	−7.338 734 920	−7.032 050 516	−10.219 934 711
200	−7.478 713 765	−7.341 186 701	−7.011 706 912	−9.183 166 843
300	−7.478 331 289	−7.343 563 794	−7.163 415 099	−8.573 938 957
400	−7.478 183 066	−7.342 453 718	−7.184 330 746	−8.522 021 325
450	−7.478 247 701	−7.343 891 621	−7.098 568 111	−8.497 641 762
500	−7.478 231 416	−7.344 161 611	−7.127 510 836	−8.495 039 086
550	−7.478 163 737	−7.344 412 016	−7.198 626 209	−8.488 917 246
650	−7.478 144 212	−7.344 961 821	−7.209 320 748	−8.485 418 119
775	−7.478 090 347	−7.345 686 536	−7.281 643 459	−8.477 869 989
900	−7.478 106 740	−7.347 166 237	−7.264 466 168	−8.471 368 155
Ritz[2]	−7.478 060 324	−7.354 098 369	−7.318 530 665	
Temple[3]	−7.478 176			

Similar tests are presented for the He atom using energy optimized Lehmann eigenvector basis sets. Also in this case, the $x_1(\varepsilon_1) > \varepsilon_2$ condition is fulfilled in all cases. The PM lower bound to the ground state energy (second column) is superior to the Lehmann lower bound (last column), generated using the pole $\rho = \varepsilon_2^-$.

Table S3: He atom ground state energy lower bound: Test for the validity of the condition that $x_1(\varepsilon_1) \geq \varepsilon_2$ for the He atom for different variance-optimized basis sets and Lehmann eigenvectors. The notation is as in Tables S1 and S2. (See Table 2 for the ε_n values used.) The last column gives the Lehmann lower bounds.

L	$x_1\left(\varepsilon_2^-\right)$ [E_h]	λ_2 [E_h]	$x_1(\varepsilon_1)$ [E_h]	$\tau(\varepsilon_2^-)$ [E_h]
20	−2.913 900 672	−0.701 229 770	−1.658 160 048	−3.163 875 676
50	−2.904 005 371	−1.583 310 706	−1.702 486 068	−2.922 294 913
100	−2.903 758 953	−1.841 993 018	−1.846 510 861	−2.907 185 738
150	−2.903 731 133	−1.982 594 206	−1.982 751 346	−2.905 210 714
200	−2.903 729 713	−2.004 193 398	−2.004 310 795	−2.904 819 525
300	−2.903 726 796	−2.012 966 252	−2.013 133 351	−2.904 369 594
400	−2.903 725 983	−2.017 047 976	−2.017 202 116	−2.904 320 053
ε_{ref}	−2.903 724 377	−2.145 974 046		

References

(1) Pollak, E.; Martinazzo, R. Lower Bounds for Coulombic Systems. *J. Chem. Theory Comput.* **2021**, *17*, 1535–1547.

(2) King, F. W. High-Precision Calculations for the Ground and Excited States of The Lithium Atom. *Adv. At., Mol., Opt. Phys.* **1999**, *40*, 57–112.

(3) Lüchow, A.; Kleindienst, H. Accurate upper and lower bounds to the ^{2}S states of the lithium atom. *Int. J. Quantum Chem.* **1994**, *51*, 211–224.
